# Planning for predictable emergency admissions to improve patient access and flow

**DOI:** 10.62675/2965-2774.20260175

**Published:** 2026-01-28

**Authors:** Airton Leonardo de Oliveira Manoel, Kathryn Chalklin, Elizabeth Butorac, Mary Copeland, Sandro B. Rizoli, Sonya Canzian, Andrew Baker

**Affiliations:** 1 Hamad General Hospital Hamad Medical Corporation Doha Qatar Trauma Intensive Care Unit, Hamad General Hospital, Hamad Medical Corporation - Doha, Qatar.; 2 St. Michael's Hospital Ontario Canada Trauma & Neurosurgery Program, St. Michael's Hospital - Ontario, Canada.; 3 St. Michael's Hospital Trauma & Neurosurgery Intensive Care Unit Ontario Canada Trauma & Neurosurgery Intensive Care Unit, St. Michael's Hospital - Ontario, Canada.; 4 St. Michael's Hospital Trauma & Neurosurgery Ward Ontario Canada Trauma & Neurosurgery Ward, St. Michael's Hospital- Ontario, Canada.; 5 Trauma Program at Hamad Medical Corporation Doha Qatar Trauma Program at Hamad Medical Corporation - Doha, Qatar.

**Keywords:** Inpatients, Length of stay, Patient discharge, Patient transfer, Quality improvement, Emergency service, hospital, Patient outcome assessment, Hospitals, Intensive care units

## Abstract

**Objective:**

To implement and evaluate a new inpatient flow process designed to reduce overnight transfers, decrease Emergency Department length of stay, and improve overall hospital throughput.

**Methods:**

A two-phase quality improvement initiative was conducted using the DMAIC (Define, Measure, Analyze, Improve, Control) framework. In Phase 1, "flow beds" were introduced to minimize overnight patient transfers and enhance staff and patient experiences. Phase 2 implemented an early morning transfer process from the trauma and neurosurgery intensive care unit to the trauma and neurosurgery inpatient ward, aligning intensive care unit capacity with anticipated daily demand. The effectiveness of the interventions was assessed using a before-and-after study design.

**Results:**

Flow beds were successfully created in over 85% of eligible cases, reducing overnight transfers to alternate units by more than 50%. Emergency Department length of stay and decision-to-admit times improved by 25% and 43%, respectively, at the 90th percentile (p < 0.05). Total inpatient length of stay decreased by 11% (approximately 1 day). In Phase 2, early trauma and neurosurgery intensive care unit discharges occurred in 50% of cases, eliminating surgical cancellations due to intensive care unit bed shortages during the study period.

**Conclusion:**

Proactive inpatient flow strategies focused on demand-capacity alignment significantly improved Emergency Department and inpatient metrics, while reducing intensive care unit-related surgical delays.

## INTRODUCTION

The demand for specialized care, including trauma, neurosurgical, and critical care services, is growing exponentially worldwide.^(1–5)^ This demand gallops faster than the average for all health services,^(6–9)^ creating significant challenges in delivering timely, high-quality care.^(10–12)^

The complex needs of trauma and neurosurgical patients present unique challenges across the continuum of care.^(1,10,13,14)^ Optimal outcomes are most consistently achieved when these patients are cared for in designated level 1 trauma centers and high-volume neurosurgical programs.^(1,3,5,11,12,15,16)^ However, delays in treatment or in transfer from the Emergency Department (ED) to the appropriate unit are associated with adverse outcomes, including increased risk of morbidity and mortality.^(6,8,9)^ In resource-constrained environments, fixed bed capacity can limit responsiveness to unplanned admissions. Therefore, a proactive and coordinated patient flow strategy is essential to ensure timely access to definitive care and to optimize outcomes for this patient population.

### The local problem

More than 50% of the time, the Trauma and Neurosurgery (TN) Program at St. Michael's Hospital in Toronto, Canada, receives five or more unplanned ED admissions overnight. These patients are best managed within the specialized TN Program either on the inpatient ward or in the trauma and neurosurgery intensive care unit (TN-ICU), depending on clinical need. However, to accommodate admissions, less acute TN patients are frequently relocated to alternative units during the night. This reactive mobilization disrupts continuity of care, increases the workload of night staff, and contributes to dissatisfaction among both patients and clinical teams.

Due to high, sustained demand for specialized services, the TN Program frequently operates at full capacity, with inpatient units and the TN-ICU often reaching 100% occupancy. As a result, elective surgical procedures may be delayed or cancelled, and the admission of patients from the ED is frequently impeded. This contributes to overcrowding, which poses significant risks to the quality of care and patient safety.^(10,11)^

During daytime hours, ED admissions and scheduled surgeries are typically accommodated through routine discharges. However, by early afternoon, both the TN ward and TN-ICU are commonly at full occupancy, leaving no capacity to accommodate overnight admissions without relocating less acute patients to alternative units.

### Intended improvement

The first phase of the initiative focused on improving patient flow from the ED to the TN inpatient ward. The primary goals of this phase were:

1. To reduce the number of patient transfers from the TN inpatient ward to other units between 10 p.m. and 6 a.m., thereby creating capacity for ED admissions.
2. To shorten the time interval from the decision-to-admit (DTA) to the ED to the actual transfer to the TN inpatient ward (DTA-Bed).
3. To decrease the overall length of stay (LOS) in the ED for trauma and neurosurgical patients.
4. To improve the patient experience by proactively and coordinately managing overflow.

The second phase of the project targeted patient flow from the TN-ICU to the TN inpatient ward. The specific objectives of this phase were to:

1. To ensure the discharge of two patients from the TN inpatient ward by 9 a.m. each weekday.
2. To facilitate the transfer of two patients from the TN-ICU to the TN inpatient ward by 10 a.m. each weekday.
3. To reduce the number of elective surgical cancellations attributable to the unavailability of TN-ICU beds.

## METHODS

### Setting

St. Michael's Hospital is a large urban academic teaching hospital fully affiliated with the University of Toronto. It serves as one of two designated level 1 trauma centers in Toronto and is among twelve adult neurosurgical centers in the province of Ontario. The TN Program encompasses a comprehensive range of services, including emergency care, surgery, diagnostic imaging, interventional radiology, trauma and neurocritical care, inpatient ward management, and an outpatient clinic.

The TN inpatient ward accommodates approximately 3,000 admissions annually and consists of 49 care beds with a nurse-to-patient (N:P) ratio of 1:4, alongside 4 intermediate care beds staffed at a 1:2 N:P ratio. The inpatient ward admits an average of 8 - 9 patients per day, and occasionally receives up to 15 patients overnight (10 p.m. - 6 a.m.). The program includes a dedicated closed-model 19-bed TN-ICU, with a 1:1 N:P ratio, which is staffed by an intensivist 24 hours a day, 7 days a week. The TN-ICU averages two to three admissions per day, totaling over 600 admissions per year.

In addition, the TN Program performs an average of three scheduled neurosurgical procedures per day, including brain tumor resection, complex neurovascular procedures, and major spinal surgery. It also manages a high volume of multiply-injured trauma patients, admitting approximately 70 - 80 such cases monthly, or roughly 800-900 cases per year.

### Research Ethics Board

Research Ethics Board (REB) approval was not sought for this project for two primary reasons. First, in accordance with the Tri-Council Policy Statement: Ethical Conduct for Research Involving Humans (second edition), the principal guideline for human research in Canada, "REB review is not required for research that relies exclusively on secondary use of anonymous information, so long as the process of data linkage or recording or dissemination of results does not generate identifiable information". Second, the same policy clarifies that quality improvement initiatives "when used exclusively for assessment, management or improvement purposes, do not constitute research and do not fall within the scope of REB review".^(17)^

### Existing flow process

Initially, the TN Program lacked a formal process to match patient demand with available bed capacity. As a result, patient flow was managed reactively rather than through anticipatory and coordinated planning. On most nights, patients were transferred from the TN inpatient ward to alternative units to create space for new ED admissions. These unplanned overnight transfers increased workload for night staff and led to dissatisfaction among both patients and healthcare providers.

Emergency Department patients who had been admitted were frequently unable to be transferred to TN ward overnight, resulting in prolonged ED LOS and additional demand on the day shift admissions. It was common for two to three admitted patients to remain in the ED awaiting transfer. The absence of a standardized process for prioritizing admissions and assigning beds further exacerbated delays. Without an established transfer hierarchy, ED admissions, TN-ICU transfers, and scheduled surgical cases all competed for the same limited number of ward beds. As a result of these flow constraints, the first TN-ICU-to-ward transfer often occurred after 11 a.m., delaying post-operative admissions to the TN-ICU and contributing to inefficiencies across the continuum of care.

### The quality improvement intervention

The quality improvement intervention focused on two specific problem areas to create efficiencies: (1) overnight admissions from the ED to the TN ward; and (2) transfer process from the TNI-CU to the TN ward.

In the first phase, the TN Program needed to develop an approach to accommodate overnight admissions from the ED. The core change underlining the first phase was the development of "flow beds" to decrease overnight transfers. This phase began on November 19^th^, 2012, and was followed by a second phase, during which the main change was an early-morning transfer process between the TN-ICU and the TN inpatient ward. This was achieved by proactively creating capacity to match expected demand.

### Phase 1: trauma and neurosurgery flow beds - create capacity

The flow team, established under the program's executive leadership, included the clinical leader managers of the TN ward and TN-ICU, a corporate patient flow specialist, the TN business manager, and the TN charge nurses. The flow specialist was responsible for tracking key performance metrics and disseminating relevant data to the team. The clinical leader, managers, business manager, and charge nurses collaborated with other clinical units to support implementation efforts.

The planning process began in April 2012 with a series of engagement meetings with TN inpatient staff to identify opportunities to improve patient flow. During these meetings, nursing staff consistently reported that transferring patients to other units during the night was a significant source of dissatisfaction. To better understand the problem, the flow team analyzed patient-level data and found that, on over 80% of nights, the TN Program received two or more admissions from the ED. This insight led to the concept of proactively creating two "flow beds" each day to accommodate expected overnight admissions.

**Figure 1:**
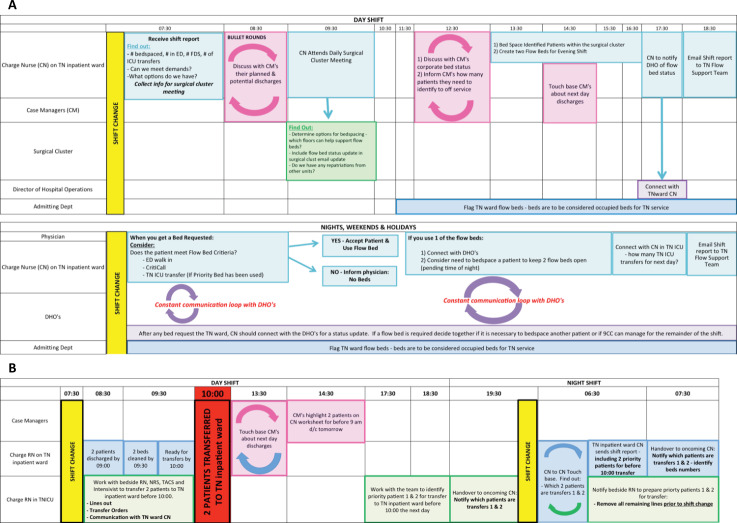
(A) The Trauma and Neurosurgery Flow Beds Project - Swim Lanes representation of the new flow process to create capacity matching demand in the trauma and neurosurgery inpatient ward (phase 1). (B) Smooth transitions by proactive planning (phase 2). The figure represents the new flow strategy adopted in phase two of the project.

The proposal, known as the Trauma and Neurosurgery Flow Beds Project, was approved by the corporate patient flow committee in September 2012 and formally launched in November 2012. The intervention involved transferring two lower-acuity patients from the TN ward to alternate units during the day shift, thereby creating two empty beds for anticipated ED admissions during the night. A visual summary of the required communication processes for successful implementation is shown in figure 1A. In parallel, the TN Program introduced an electronic shift report distributed every 12 hours by the ward charge nurse that identified the designated flow beds and the number of overnight ED admissions. This report became a standardized communication tool between charge nurses and served as a key data source for monitoring and evaluating the intervention.

DHO - director of hospital operations.

To avoid a negative impact on other inpatient units across the institution, three guiding principles were adopted:

1. Avoid TN encroachment on other services: TN patients would be repatriated from other units, and elective TN surgeries would be put on hold/cancelled when necessary to avoid surgical cancellations in other programs.
2. Manage within existing resources: other inpatient units would not be asked to open unbudgeted beds or flex up to create flow beds.
3. Respect organizational capacity limitations: if institutional capacity were at 100%, flow beds would not be created that day.

The TN Program engaged the other surgical programs from the beginning of the project via the ‘surgical cluster’, which is a daily bed management meeting. Much of the feedback received was consistently positive. Themes from the surgical cluster included increased communication within the organization, optimized patient care, and improved patient flow.

At both the corporate and the local level, previous efforts to enhance patient flow had been attempted with limited success. At the onset of this project, some staff viewed ‘creating’ flow beds as an additional task that would only positively impact the night shift.

After a few months, the initiative's benefits became clear, and the culture around patient flow began to change. Obtaining staff buy-in was crucial, as this group would carry the project into a sustainability phase and eventually into common practice. Within 4 weeks of the Trauma and Neurosurgery Flow Beds Project launch, the overnight ED admissions were successfully accommodated in inpatient beds before the start of the day shift. Decision-making and planning for scheduled surgeries and in-house transfers were simplified, without the added pressure of admitted patients in the ED awaiting beds. This also allowed first-morning discharges to be assigned to the TN-ICU and marked the start of the second phase of the flow project.

### Phase 2: smooth transitions by proactive planning

While the TN Program did very well at creating capacity for ED patients, it was also important to plan for morning ward discharges to accommodate transfers from the TN-ICU (Figure 1B). An interdisciplinary brainstorming session was held to identify the processes required to discharge two patients from the TN inpatient ward by 9 a.m. each weekday. This would allow 1 hour to prepare the ward beds to accommodate two transferring patients from the TN-ICU by 10 a.m.

The planning began in the TN-ICU during afternoon sign over rounds with the intensivist and ICU charge nurse identifying two priority patients for transfer to the ward for the next morning (Figure 1B). The patients were visually flagged on the unit census board with two buttons saying #1 and #2. Laminated cards were given to the bedside nurses during the night shift as a cue that their patient would be one of the two priority transfers. It became the responsibility of the night shift to ensure that priority transfer patients were ready for transfer before shift change. The night shift nurses were responsible for discontinuing invasive devices, ensuring intravenous medications were switched to oral formulations when applicable, and writing electronic transfer orders.

Since identified priority transfer candidates were often neurosurgical patients who required brain imaging such as computed tomography (CT) scanning prior to discharge from TNI-CU, a new process was developed with the Diagnostic Imaging Department to accommodate overnight imaging for priority transfer patients. It was agreed that CT and magnetic resonance imaging would accommodate up to two TN-ICU patients between 4 a.m. and 6 a.m. to ensure imaging results would be available for 6 a.m. rounds in the TN-ICU. It became the expectation that these patients were ready for transfer to the ward by 7:30 a.m.

Proactive planning was also occurring on the ward. During the day shift, the case managers and the ward charge nurse were required to identify two patients to be discharged home before 9 a.m. the next morning. This information was communicated to the night shift to help ensure patients were ready as quickly as possible the next morning.

At the end of the night shift, a shift report from the TN inpatient ward was circulated, including the bed assignment for the two TN-ICU transfers. In the TN-ICU, the team focused on having two patients ready for transfer before 10 a.m. This allowed 1 hour for terminal bed cleaning in preparation for an incoming TN-ICU admission.

### Methods of evaluation - how do we know that a change is an improvement?

A) Process measures:

- Phase 1: the percentage of time the flow beds were created.
- Phase 2: the percentage of time the TN-ICU discharged two patients before 10 a.m.

B) Outcome measures:

Phase 1:

- The number of overnight transfers.
- ED LOS and time from ED admission to TN ward bed (DTA-Bed).
- Total inpatient LOS.

Phase 2:

- Number of elective neurosurgical procedure cancellations.
- Total inpatient LOS.

### Statistics analysis

The data collected were divided into three time periods:

#### Pre-project implementation

P1) From November 19^th^, 2011 to April 30^th^, 2012.

P2) From May 1^st^, 2012 to November 18^th^, 2012.

#### Post-implementation phase

P3) From November 19^th^, 2012 to April 30^th^, 2013.

Time periods P1 and P3 were the same date ranges over two consecutive years. Due to the seasonal variation, particularly with trauma cases, it was important to look at the same time periods to maintain consistency.

The data from P3 were compared with those from P1 for DTA-Ward and ED LOS. Both metrics were tested using the Log-Rank and Wilcoxon tests to determine significance at the 5% level. Statistical Analytic Software was used to perform the data analysis.

## RESULTS

### Phase one: trauma and neurosurgery flow beds - create capacity

**Process measures:** two TN flow beds were created over 85% of the time during the project period (P3). The remaining 15% of the time, the institution was at or above 100% occupancy, with no available beds for TN to use.

**Outcome measures:** during the planning phase (pre-project implementation), 94 TN patients had been transferred to other units during the night shift (7:30 p.m. - 7:30 a.m.) to accommodate ED admissions, which corresponded to 0.4 transferred patient per night (Table 1). Eighty overnight transfers (10 p.m. - 6 a.m.) occurred in the same pre-project period. Following the flow beds implementation, the number of patients transferred during the night shift (7:30 p.m. - 7:30 a.m.) and overnight (10 p.m. - 6 a.m.) decreased to 43 and 30, respectively. This represents a 48% reduction in overnight transfers.

**Table 1:** Number of overnight transfers from the trauma and neurosurgery ward to an alternative unit

	Pre-implementation Apr 1/12 - Nov 18/12	Post-implementation Nov 19/12 - May 1/13
Number of patients - night shift (7:30 p.m. - 7:30 a.m.)	94	43
Total days in the period	232	168
Total transferred patients	0.40 patient/night shift	0.25 patient/night shift
Number of patients - Overnight (10 p.m. - 6 a.m.)	80	30
Total days in the period	232	168
Total bed spacing	0.35 patient/night	0.18 patient/night

**Figure 2:**
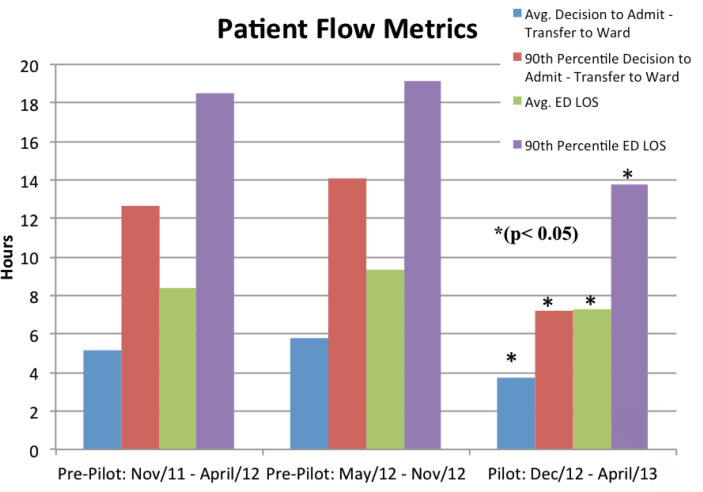
Emergency Department patient flow metrics.

Since capacity was created on the TN inpatient ward, the patient flow metrics in the ED were also improved. The average time from DTA in the ED to transfer to the TN inpatient ward was reduced by 28% and 43% at the 50^th^ and 90^th^ percentiles, respectively. Corporately, St. Michael's Hospital has a patient flow target for the DTA-Ward within 4 hours. The TN Program's average DTA-Ward was reduced from 5.8 hours to 3.7 hours (p < 0.05). Total ED LOS was reduced by 18% and a total of 755 ED hours were saved (Table 2). Average ED LOS was reduced by 14% (about 1.2 hour) and 26% (about 4.8 hours) at the 90th percentile (Table 2 and Figure 2).

**Table 2:** Emergency Department patients’ flow metrics

	ED volume	ED avg LOS (hours)	ED 90P LOS (hours)	Avg DTA to bed (hours)	90P DTA to bed (hours)
Pre-Pilot: Nov 19/11 - Apr 30/12	497	8.39	18.53	5.17	12.63
Pre-Pilot: May 1/12 - Nov 18/12	618	9.31	19.17	5.79	14.08
Pilot: Nov 19/12 - Apr 30/13	471	7.25	13.73	3.71	7.18

ED Avg. LOS - Emergency Department average length of stay; ED 90P LOS - Emergency Department 90% percentile length of stay; Avg DTA to bed - average time from decision to admission in Emergency Department to bed in trauma and neurosurgery ward; 90P DTA to bed - 90% percentile time from decision to admission in Emergency Department to bed in trauma and neurosurgery ward.

ED - Emergency Department.

We hypothesized that improving flow would decrease inpatient LOS. A 6.4%-reduction (1/2 day) was observed in the first quarter, and an 11.2%-reduction (1 day) was seen in the second quarter, after the project rolled out.

**Balancing measures:** one surgical cancellation occurred in the General Surgery Department due to a TN patient on that unit. No additional delays or surgical cancellations were reported.

### Phase 2: smooth transitions by proactive planning

**Figure 3:**
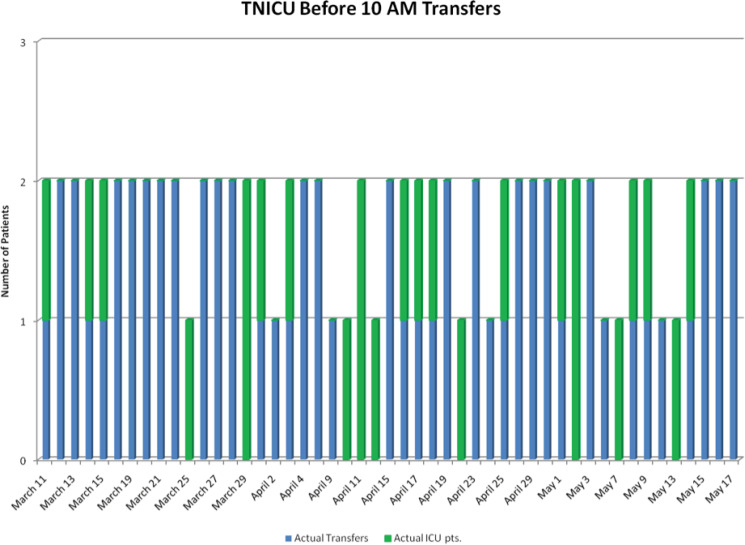
Transfer from trauma and neurosurgery-intensive care unit to trauma and neurosurgery inpatient ward before 10 a.m. The green bars represent demand (if the green bar touches the #1 line, it indicates that the trauma and neurosurgery-intensive care unit had only one patient stable enough for transfer). An instance where the green bar is covered by a blue bar indicates patients who met the target of being transferred to the trauma and neurosurgery ward before 10 a.m. Visible green bars indicate days when transfer demand was not met.

The TN-ICU was able to transfer two patients before 10 a.m. approximately 50% of the time (Figure 3). One patient was transferred before 10 a.m. 25% of the time, leaving 25% without a transfer before 10 a.m. This reduced surgical cancellations, resulting in none due to bed availability during the project period.

TN-ICU - trauma and neurosurgery-intensive care unit; ICU - intensive care unit.

Since the implementation of the project, there has been increased collaboration in patient care planning between the TN-ICU and TN ward. This is a result of enhanced communication among charge nurses through the formal electronic shift report, which has become an open channel for communication among the TN ward, TN-ICU, and program leadership.

## DISCUSSION

The authors successfully redesigned and implemented a new inpatient flow process across the trauma and neurosurgery units by introducing a proactive patient movement strategy to create capacity for unplanned overnight Emergency Department admissions. This approach effectively reduced the need for overnight transfers. Prior to the intervention, high volumes of overnight ED admissions to the TN inpatient ward often necessitated transferring less acute TN patients to alternative units during the night to accommodate new arrivals. This reactive mobilization led to staff dissatisfaction and increased the workload for the night shift. Furthermore, patients frequently experienced prolonged ED stays, with average wait times ranging from 8 to 10 hours before being transferred to the TN ward.

Following the implementation of the intervention, the average time from DTA to ward transfer was reduced from 5.8 hours to 3.7 hours (p < 0.05), and the 90th percentile DTA-Bed time improved from 14.1 to 7.2 hours, surpassing Ontario's provincial benchmark of 8 hours at the 90th percentile for admitted ED patients. Corporately, St. Michael's Hospital has adopted a more stringent internal target of completing DTA-to-ward transfers within 4 hours. This aligns with recommendations from the Canadian Association of Emergency Physicians and Ontario Health's performance framework, and it served as a guiding benchmark for the design and evaluation of our intervention.

Emergency Department overcrowding has become an increasingly common challenge in North American hospitals. A 2002 US national report found that over 90% of large American hospitals were operating above capacity,^(10)^ highlighting a system-wide strain. This issue is particularly concerning in the context of Ontario's universal healthcare system, where the Ontario Health Insurance Plan serves as the sole payer for a population of approximately 13.5 million. Emergency Department overcrowding leads to delays in diagnosis and treatment, reduces the quality of care, and compromises patient safety.^(11,12,15,16)^

By proactively transferring two patients to alternate units during the day shift to create capacity for overnight admissions, the number of nighttime transfers and the ED LOS decreased significantly. Numerous studies have demonstrated that shorter ED LOS is associated with improved patient outcomes.^(2,4,8,9)^ Acutely ill patients benefit from timely transfer to specialized inpatient wards or, in some severe cases, to the ICU. Notably, Chalfin et al., analyzing a large North American administrative database, found that delays in ED-to-ICU transfer were associated with longer hospital LOS and higher ICU and hospital mortality rates.^(7,8)^

The second phase of the project focused on establishing early morning transfers from TN-ICU to the inpatient ward. Prior to implementation, morning discharges from TN-ICU typically occurred after 11:30 a.m., resulting in delays in elective surgery cases and, occasionally, surgical cancellations. Surgical cancellations reduce operating room efficiency and result in wasted resources.^(10,12,18)^ From the perspective of patients and their families, the cancellation of a planned surgery can cause significant anxiety, frustration, and anger.^(1,13,14,19,20)^ Although the pre-intervention cancellation rate for elective neurosurgical procedures was low (0.5 - 0.6%), no elective cases were cancelled due to TN-ICU bed unavailability during the project period. Importantly, delays in ICU-to-ward transfers can also increase hospital costs, prolong ICU LOS, and expose patients to additional risks, including hospital-acquired infections.

### Lessons learned

The flow projects led to several important and lasting improvements. Most notably, the introduction of a standardized electronic TN charge nurse summary report significantly enhanced communication and collaboration. Prior to the intervention, coordination between the TN-ICU and the TN inpatient ward was inconsistent. The electronic shift report formalized early-morning communication among charge nurses, enabling shared situational awareness and proactive planning of patient movement. Since the implementation of the project, the collaboration in patient care planning between the TN-ICU and TN inpatient ward has significantly increased, strengthening interdisciplinary teamwork and enhancing continuity of care.

Notably, the flow strategies developed during this project have been sustained within the TN Program well beyond the initial implementation period. Key components, such as early ICU-to-ward transfers and proactive flow bed planning, have since been adopted by other surgical and critical care units across the institution. Although the original data span 2012 - 2013, the principles of early planning, multidisciplinary engagement, and demand-capacity matching remain highly relevant and continue to guide current hospital flow strategies.

### Limitations

This study has several limitations. First, as a quality improvement initiative conducted at a single academic trauma and neurosurgical center, the findings may not be generalizable to other institutions, particularly those in non-tertiary or community settings. The unique structure and resource availability at St. Michael's Hospital, including dedicated trauma and neurosurgical inpatient units and intensive care, may not be replicable in all hospitals.

Second, the intervention was implemented and evaluated between 2012 and 2013. Although the strategies remain in place and have influenced institutional practices beyond the study period, the data presented reflect a historical time period. They may not fully capture current trends in Emergency Department utilization, hospital occupancy, or case complexity.

Third, the primary focus was on system-level process and outcome measures (e.g., ED LOS, inpatient transfers, surgical cancellations). While these are highly relevant for operational efficiency, we did not collect patient-level clinical outcomes such as morbidity, mortality, or patient-reported experience measures. Future evaluations could explore whether improved flow metrics translate into measurable improvements in patient outcomes and satisfaction.

Fourth, the study did not include a formal cost analysis. While the intervention was designed to operate within existing resources, understanding its economic impact, both in terms of cost avoidance and potential resource reallocation, would provide additional value for system-level decision-making.

Finally, sustainability and implementation challenges are inherent to any quality improvement initiative. Securing staff buy-in, standardizing communication tools, and maintaining adherence to early discharge targets required ongoing leadership engagement and cultural change. While the program demonstrated durability within the TN units, broader adoption across the institution involved adjustments to local workflows and competing service priorities. These contextual factors may limit scalability without tailored implementation strategies.

## CONCLUSION

Proactively planning for predictable demand, combined with targeted strategies for demand-capacity matching, can significantly improve patient flow across hospital inpatient units. This quality improvement initiative demonstrated that process mapping and earlier discharge planning can reduce unnecessary patient movement, enhance communication among clinical teams, and optimize the patient experience. At St. Michael's Hospital, the implementation of flow beds and early intensive care unit-to-ward transfers led to measurable improvements, including a reduction in Emergency Department length of stay, total inpatient length of stay, and overnight transfers. These findings highlight the importance of structured, interdisciplinary approaches to flow optimization in high-acuity environments such as trauma and neurosurgical care.

## Data Availability

Data is available on demand from referees.

## References

[1] MacKenzie EJ, Rivara FP, Jurkovich GJ, Nathens AB, Frey KP, Egleston BL (2006). A national evaluation of the effect of trauma-center care on mortality. N Engl J Med.

[2] Herring AA, Ginde AA, Fahimi J, Alter HJ, Maselli JH, Espinola JA (2013). Increasing critical care admissions from U.S. emergency departments, 2001-2009. Crit Care Med.

[3] McNeill L, English SW, Borg N, Matta BF, Menon DK (2013). Effects of institutional caseload of subarachnoid hemorrhage on mortality: a secondary analysis of administrative data. Stroke.

[4] Wunsch H, Angus DC, Harrison DA, Collange O, Fowler R, Hoste EA (2008). Variation in critical care services across North America and Western Europe. Crit Care Med.

[5] Tepas JJ, Pracht EE, Orban BL, Flint LM (2013). High-volume trauma centers have better outcomes treating traumatic brain injury. J Trauma Acute Care Surg.

[6] Richardson JD, Franklin G, Santos A, Harbrecht B, Danzl D, Coleman R (2009). Effective triage can ameliorate the deleterious effects of delayed transfer of trauma patients from the emergency department to the ICU. J Am Coll Surg.

[7] Parker A, Wyatt R, Ridley S (1998). Intensive care services; a crisis of increasing expressed demand. Anaesthesia.

[8] Chalfin DB, Trzeciak S, Likourezos A, Baumann BM, Dellinger RP, DELAY-ED study group (2007). Impact of delayed transfer of critically ill patients from the emergency department to the intensive care unit. Crit Care Med.

[9] Rincon F, Mayer SA, Rivolta J, Stillman J, Boden-Albala B, Elkind MS (2010). Impact of delayed transfer of critically ill stroke patients from the Emergency Department to the Neuro-ICU. Neurocrit Care.

[10] Trzeciak S, Rivers EP (2003). Emergency department overcrowding in the United States: an emerging threat to patient safety and public health. Emerg Med J.

[11] Taylor TB (2001). Threats to the health care safety net. Acad Emerg Med.

[12] Derlet RW, Richards JR (2000). Overcrowding in the nation's emergency departments: complex causes and disturbing effects. Ann Emerg Med.

[13] Rabinstein AA, Lanzino G, Wijdicks EF (2010). Multidisciplinary management and emerging therapeutic strategies in aneurysmal subarachnoid haemorrhage. Lancet Neurol.

[14] Diringer MN, Edwards DF (2001). Admission to a neurologic/neurosurgical intensive care unit is associated with reduced mortality rate after intracerebral hemorrhage. Crit Care Med.

[15] Derlet RW, Richards JR (2002). Emergency department overcrowding in Florida, New York, and Texas. South Med J.

[16] Gordon JA, Billings J, Asplin BR, Rhodes KV (2001). Safety net research in emergency medicine: proceedings of the Academic Emergency Medicine Consensus Conference on "The Unraveling Safety Net". Acad Emerg Med.

[17] Canadian Institutes of Health Research; Natural Sciences and Engineering Research Council of Canada; Social Sciences and Humanities Research Council of Canada (2014). Tri-Council Policy Statement: Ethical Conduct for Research Involving Humans (TCPS 2).

[18] Schofield WN, Rubin GL, Piza M, Lai YY, Sindhusake D, Fearnside MR (2005). Cancellation of operations on the day of intended surgery at a major Australian referral hospital. Med J Aust.

[19] Tait AR, Voepel-Lewis T, Munro HM, Gutstein HB, Reynolds PI (1997). Cancellation of pediatric outpatient surgery: economic and emotional implications for patients and their families. J Clin Anesth.

[20] Ivarsson B, Kimblad PO, Sjöberg T, Larsson S (2002). Patient reactions to cancelled or postponed heart operations. J Nurs Manag.

